# Financial burden and quality of life among early‐onset colorectal cancer survivors: A qualitative analysis

**DOI:** 10.1111/hex.12919

**Published:** 2019-07-05

**Authors:** Erica Blum‐Barnett, Sarah Madrid, Andrea Burnett‐Hartman, Shane R. Mueller, Carmit K. McMullen, Andrea Dwyer, Heather S. Feigelson

**Affiliations:** ^1^ Institute for Health Research Kaiser Permanente Colorado Aurora Colorado; ^2^ Center for Health Research Kaiser Permanente Northwest Portland Oregon; ^3^ Fight Colorectal Cancer The Colorado School of Public Health Aurora Colorado

**Keywords:** early‐onset colorectal cancer, financial burden, qualitative, quality of life, survivorship

## Abstract

**Background:**

Colorectal cancer (CRC) diagnosed at ages <50 years old (early‐onset CRC) has been increasing in the United States, resulting in a growing number of early‐onset CRC survivors who may face significant financial and quality of life (QOL) challenges.

**Objective:**

Identify themes from a patient advocate discussion about the impact of CRC on financial burden and QOL among early‐onset CRC survivors.

**Methods:**

We conducted a semi‐structured, stakeholder discussion among 14 early‐onset CRC survivors and one caregiver who were members of an advocacy group. The discussion focused on the financial and overall QOL impacts of CRC. The meeting was recorded, transcribed and coded in ATLAS.ti, using a thematic analysis approach.

**Results:**

Cancer stage at diagnosis among advocates with CRC ranged from 2 to 4; about half of the attendees had no evidence of disease, and about half were undergoing treatment. Employment (career trajectory, lost wages, health insurance/benefits, performance) emerged as the dominant theme of the financial impacts discussion. Lifestyle impacts of disease and survivorship included both emotional and physical side‐effects. Diagnosis experience, missing information about CRC treatment and side‐effects, financial stress and strain on relationships were the primary themes for the overall QOL impacts.

**Conclusion:**

Given the growing incidence of CRC in those under 50, it is particularly important for providers to be aware of these patients' financial, emotional and QOL needs, and to develop care plans that specifically address these areas of concern for early‐onset CRC survivors.

## 1 INTRODUCTION/PURPOSE

In 2019, over 140 000 cases of colorectal cancer (CRC) will be diagnosed in the United States and there is a growing number of cancer survivors who report unmet needs.^1^^,^ 2 While the incidence of CRC among older adults has been declining, the incidence of early‐onset (defined as being diagnosed under age 50) CRC is on the rise in the United States.^3^ CRC survivors of all ages may experience profound physical and emotional side‐effects, impacting their overall quality of life (QOL). The physical side‐effects of CRC include long‐term nerve damage, incontinence, problems with fertility, changes in sexual function and lifestyle changes associated with ostomy.^4^, ^5^, ^6^, ^7^, ^8^, ^9^ Emotional side‐effects result from responses to the physical side‐effects, stress, changes in relationships, confidence and changes in identity.^9^ It is possible that younger CRC survivors experience these side‐effects differently than older survivors. Further, those diagnosed with CRC at a young age may face difficult financial challenges.^10^ A systematic review of work‐related issues among young cancer survivors found that those who returned to work struggled with reduced work productivity that ultimately impacted their income.^11^ A cancer diagnosis results in high financial burden,^12^, ^13^, ^14^, ^15^, ^16^ and there is an increase in the risk of bankruptcy following a cancer diagnosis.^16^ For cancer patients who are <50 years old, the risk of bankruptcy following their cancer diagnosis is particularly high.

To address the unique needs of the growing population of early‐onset CRC patients, health systems must understand the specific challenges that these patients face. This includes both the short‐ and long‐term consequences associated with CRC and its treatment. While prior studies have examined QOL indicators in CRC survivors,^17^, ^18^, ^19^, ^20^ few studies have evaluated how CRC may specifically impact the QOL of early‐onset CRC patients. Similarly, little is known about the drivers behind the financial stress that is particularly high in those diagnosed with cancer under the age of 50.

This study examines QOL and financial impacts of a cancer diagnosis among early‐onset CRC survivors.

## 2 METHODS

### 2.1 Study setting

In December 2015, HSF, ABH, SM, AD and EBB met with a group of early‐onset CRC survivors and one caregiver who were attending a two‐day Fight Colorectal Cancer (CRC) (https://fightcolorectalcancer.org/) advocacy training. Fight CRC is a patient advocacy group that works for better policies and research for those who are impacted by CRC. The purpose of this semi‐structured meeting was for researchers to elicit qualitative feedback about primary changes in the quality of life and financial strain that early‐onset CRC patients' experience. The CRC advocates shared their experiences to help with research prioritization, and the researchers considered the Fight CRC advocates to be partners in patient engagement research. According to Hamilton, et al,^21^ ‘Patient engagement in research involves patients or their surrogates undertaking roles beyond those of traditional study participants along the continuum of the research process…’ All members of the panel provided verbal consent to participate and agreed to have the meeting recorded and transcribed.

The study is part of the Patient Outcomes Research to Advance Learning (PORTAL) network^2^ and funded as part of the Patient‐Centered Outcomes Research Institute's (PCORI) PCORnet.^22^, ^23^

### 2.2 Meeting structure

**Figure 1:**
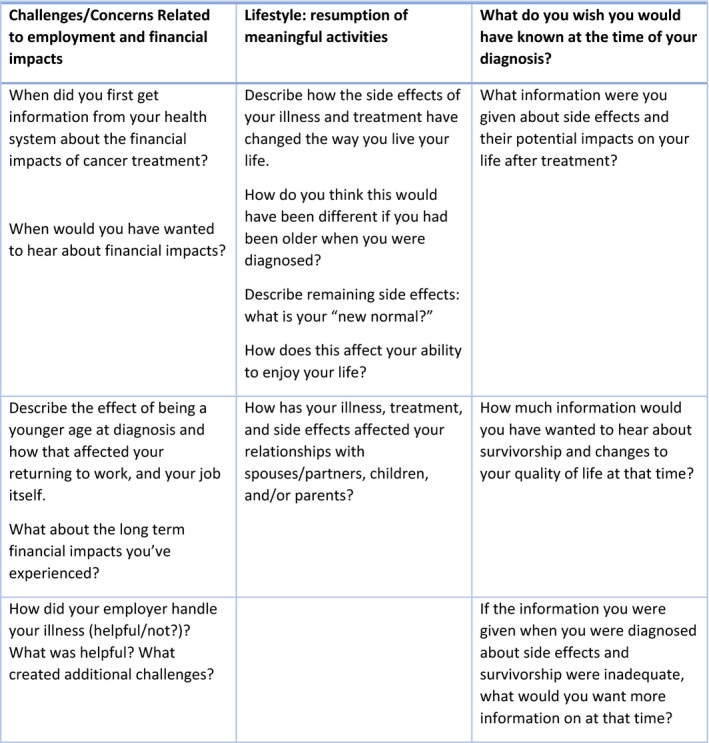
Discussion guide

Prior to the meeting, researchers specializing in CRC (HSF, ABH and AD) met to discuss how to best use the opportunity of meeting with the advocates. Based on gaps in the existing research, the researchers decided to ask the panel about concerns and challenges related to employment, financial impacts, lifestyle and resumption of meaningful activities. A researcher trained in patient engagement and qualitative research (SM) moderated and used these topics to guide the 2‐hour patient advocate meeting using focus group methods to engage all advocates in a discussion of these topics.^24^ In order to provide context for the meeting, one researcher (HSF) presented information on the research that led up to the meeting and described the demographics of a cohort of 16 000 people diagnosed with CRC.^2^ HSF explained that the purpose of the meeting was to hear directly from the advocates about research topics that were important to them that could be studied in this large cohort of CRC survivors. Starting the meeting with a presentation on the facilitators' research could have introduced a small bias, but providing context for the researchers experience and reason for the meeting was important for setting the stage to elicit feedback. The presentation was followed by a semi‐structured discussion led by the moderator, SM (See Figure 1). To foster discussion, SM used probes to solicit additional information without biasing advocate responses. Each advocate was asked to respond to ensure that all members had an opportunity to provide input about each topic. When non‐verbal communication was expressed by advocates throughout the discussion, it was noted.

The meeting recording was transcribed, and all names were masked using pseudonyms. The final transcript was reviewed for accuracy by comparing the transcript against the original recording.

### 2.3 Data analysis

#### 2.3.1 Generating codes and identifying themes

Two investigators (SM and EBB) conducted an inductive qualitative analysis of the discussion using a thematic analysis approach.^25^, ^26^, ^27^ First, the transcript was reviewed for overall understanding and first impressions.^28^ Then, the first 10 minutes of the discussion was open‐coded^26^ independently by each investigator to identify patterned responses to generate initial codes. The resulting code lists were compared and discussed, and a master code list was created that reflected the themes identified by each coder in a standardized manner.^21^

**Figure 2:**
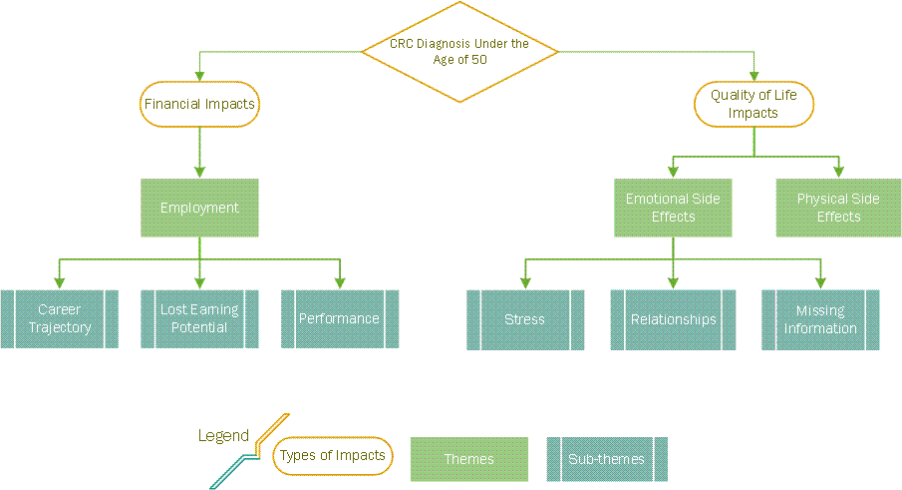
Themes identified during the qualitative analysis of meeting transcript

The transcript was then imported into ATLAS.ti (Version 8), and the final master code list was used to independently code the entire meeting. Coding was conducted using a thematic analysis approach.^27^ Consistent with this method, we iteratively compared coding, revised codes to establish agreement among analysts and recorded the evolving definitions of the existing codes, adding new codes that emerged as necessary. When there was disagreement, it was resolved by discussion and referencing of the code definitions. Over the course of the team coding process, the researchers sorted many of the codes into sub‐themes that were grouped under overarching themes. As a final step, coding was reviewed by the study team for consistency. Overarching themes that carried the most importance in relation to the research questions and contributed to the conceptual map emerged from this process (See Figure 2).

## 3 RESULTS

### 3.1 Advocate characteristics

Fight CRC patient advocates from around the United States participated in the meeting. The advocates included fourteen CRC survivors who had been diagnosed under the age of fifty and one caregiver. All of the advocates had health insurance at the time of the meeting. Additionally, all advocates were well enough to travel and were able to incur some out of pocket costs to attend. About half of the survivor advocates at the meeting reported that they had no evidence of disease, while the other half were undergoing treatment. Stage of disease at the time of diagnosis among the group ranged from Stage II to Stage IV.

### 3.2 Themes

Several sub‐themes emerged within two overarching themes: financial impacts and QOL.

### 3.3 Financial Impacts

Employment emerged as the overarching theme from the discussion of financial impacts of a CRC diagnosis. The advocates communicated that a CRC diagnosis impacted employment in four distinct areas: (a) Career trajectory; (b) Lost earning potential; (c) Health insurance/Benefits; and (d) Performance.

#### 3.3.1 Employment

##### Career trajectory

Advocates described a CRC diagnosis impacting their employment at pivotal moments in their careers. They described how CRC had interrupted their career trajectories, slowed their professional development and impacted their inability to take on additional responsibilities at work. One advocate shared the ways her Stage IV CRC diagnosis and treatment significantly disrupted her career trajectory as a university professor:

…for many of us that are in this cohort of being under 50, you’re talking about a really catastrophic hit to your career. I mean I was [in my 30s] when I was diagnosed. I was supposed to be tenure track. And now I'm not.

#### 3.3.2 Lost earning potential

Colorectal cancer resulted in lost earnings for advocates, as well as a setback in their earning potential. The diagnosis interrupts career trajectories as described above and is compounded by the cost of treatment. One advocate expressed gratitude for his continued employment as a financial analyst, but noted that complicated disability policies, insurance expenses and other outside costs related to CRC kept him at a static income level. He said:

The amount I pay for my cost‐share portion of the premium is going up every year. My deductibles and co pays and out of pocket maxes are going up each year. My salary isn't changing that much and the portion that disability pays is locked in to what my salary was two years ago and even when my employer is generous enough to give me a 1% raise, that's money I earn that reduces the disability payment so I'm basically at a static income level with all of these other costs going on. That's just the new reality.

One advocate found the concept of lost earnings to be particularly important, re‐directing the discussion on finances at one point by saying:

We talk about the nitty gritty of paying [for care and treatment] and I understand that that's important, but we have to talk about lost earnings, too. Especially at this age…

Many people save for retirement and plan financially without accounting for the possibility of catastrophic illness. Even if they do plan for the possibility, they may still spend their savings on cancer‐related costs before reaching retirement. One of the meeting advocates shared that after being diagnosed, she thought she was going to die so did not think she needed to continue saving. Another advocate echoed a similar sentiment by highlighting the natural disregard for future planning when hit with a cancer diagnosis saying:

Once you realize “I'm going to survive this,” you need to worry about how you're going to pay for everything.

#### 3.3.3 Health insurance/Benefits

Advocates described how health insurance and work benefits were inadequate to support their treatment and the resulting financial impacts that occur with early‐onset CRC. More than one advocate mentioned that prior to their diagnosis, they had been hesitant to sign up for more advanced health insurance benefits because they never thought *they* would get sick. Others said they did not sign up because of poor communication about risks from insurance companies or from the employer providing the benefits.

One advocate had been thinking about short‐term disability insurance for years because of pre‐existing asthma. Fortunately, she completed the paperwork for short‐term disability just 3 months before she was diagnosed with CRC. She said:

…if it wasn't for that, I would have had zero income. Absolutely nothing. I didn't sign up for the max so the amount that I was getting only covered my rent, so my family still had to pay the rest of my bills, but at least I still had somewhere to live, because I really would have been homeless.

Throughout the discussion, the group compared the widely varying insurance coverage costs among the advocates. One advocate highlighted the heightened cost of health insurance for an individual who is self‐employed and enrolled in a less than ideal coverage plan. In responding to a participant who noted that she paid $300 per month for insurance coverage, she said:

Three hundred a month? We're [spending] fifteen hundred a month and our care is terrible. And I… looking through the lens of cancer… I just cross my fingers because if I wanted a good plan that kept our copays down and kept our deductible down, I would be up at like twenty‐eight hundred a month because I'm not part of a group and with today's economy I see a lot more people in those shoes where people don't have employers paying for it, they don't work for the government or a school system and if you're one of those individuals, you're SOL when it comes to this stuff.

#### 3.3.4 Performance

Several advocates discussed how the physical side‐effects of disease and treatment impacted their work performance. One advocate described her experience of changed mental capacity and difficulty with performing physical tasks required when she returned to work after one year of sick leave, saying:

…before I got sick, I was an instructor when new hires came. And then when I came back to work, I faked it for 6 weeks…with cognitive dysfunction. The neuropathy. I can't sit and type all day. My attention span is not the same.

She continued, describing the way she grappled with what she perceived as co‐worker's expectations of her to perform at a ‘normal’ level despite her lived reality as a CRC survivor:

You know, and everybody's used to me being, you know, “on it.” But it's like I don't know how to explain to them… and I'm still struggling with that because I just went back to work a month ago. And they want me in the same capacity, but I don't know. It's kind of embarrassing to be honest. I don't how to say, “I'm not that person anymore.' I don't have my memory anymore. I don't… you can't come to me like you used to and ask me these questions because I don't know. And I don't know where to go research it. I just don't know…You want to still be respected but I'm not the same.

Another advocate provided an example of how the physical side‐effects made it difficult to do what was required of her and emphasized the way each survivor experiences different side‐effects:

…my pelvic girdle is very weak and it's painful and we've been working on it. I'm a physical therapist. I'm supposed to lift heavy people. It's like you don't even realize all of the problems that are coming up. And each person as you talk here… everybody is different.

### 3.4 QOL impacts

Advocates described how a CRC diagnosis and the subsequent treatment had significant impacts on their QOL due to both emotional and physical side‐effects. Emotional side‐effects were particularly emphasized when participants brought up their (a) *stress* associated with financial burdens of survivorship, (b) *relationship* changes and strain and (c) *missing information*/*resources from providers*.

#### 3.4.1 Emotional side‐effects

##### Stress

While there are many different sources of stress for all cancer survivors, meeting advocates emphasized the emotional stress that came along with the financial burdens of being diagnosed with CRC at a younger age. One advocate said:

So, we're talking about the stress of being sick, yeah that's there because I was sick for real but the financial aspect of it is just so stressful and no one tells you… You're already… you're going through so much. And not to have any money on top of it? It was the worst time of my life.

##### Strained relationships

A cancer diagnosis often impacts not only the patient, but also entire families because of the stresses and other emotional impacts of caregiving. Multiple advocates spoke about the ways that their need for care and their physical side‐effects impacted intimate relationships in their lives. One woman described the way her cancer diagnosis led to the disintegration of her marriage:

The hard part was I was married for 24 years and my husband decided my cancer ruined his life and my ostomy disgusted him …

The advocates described their roles as parents, partners and caregivers for other family members and grappled with the perceived expectations to fulfill those roles for other people. One participant described her experience as someone who needed to be cared for at a point in her life when she is expected to care for others:

I'm married, my husband is also my caregiver. And how do you shift back and forth between a caregiver and being romantic? And also being a spouse and a parent… Those are really difficult things to sort of try and juggle. …my kids have seen me in some of my most vulnerable moments… and all of a sudden you're the one that needs to be taken care of.

##### Missing information about ‘what to expect’

Advocates expressed frustration about the lack of information from their providers and insurance companies regarding the impacts of their diagnosis and treatment. They emphasized the need for more resources and better communication about existing resources specific to early‐onset CRC. Specifically, they sought more information about what to expect from chemotherapy and ostomy; impacts of disease and treatment on job performance, relationships and sexual function; dietary changes and what to eat during the cancer care continuum; and the different types of financial impacts. One participant wished she had access to more information about sexual side‐effects of disease and treatment. She said:

…for a lot of people under 50, colon and BM is hard enough to talk about but sexual dysfunction is another thing and that's really hard to talk about. And those are some huge side effects as well that people struggle with. And it's hard to get answers. You know I said to my radiation doctor‐ I asked him about sexual dysfunction and he told me to talk to the techs in the lab. [Laughter, murmurs] And so you know, it's really hard.

#### 3.4.2 Physical side‐effects

Physical side‐effects of CRC and treatment were described throughout the discussion and often seemed to be a point of comradery among the participants in the room. One participant noted a benefit of being in this group of advocates was the ability to speak openly about physical side‐effects, which were often considered taboo in other settings. Participants implied that there is a layer of emotional shame that coincided with the stigmatized physical side‐effects specific to CRC.

The list of physical side‐effects that surfaced among participants during the discussion was long: the short‐term and lasting effects of chemo and radiation, adjusting to life with ostomy, sexual dysfunction, problems with bowel and bladder control, and neuropathy. The following dialog captured agreement on the experience of adapting to a new life with the physical side‐effects of survivorship:

P1: I don't think the cancer's going to come back and get me, but the side effects might get me.

P2: Amen

P1: I just roll with it and that's my life now. Part‐time job, going to the doctors, and surgery.

P3: Some job.

P1: Yeah, it's a great job.

P4: That you didn't apply for.

The way that participants equated the physical side‐effects to having a job highlights the scale of the impacts, the time they take up and the unwanted responsibility of adjusting to their new lives.

## 4 DISCUSSION

While survivorship issues are well documented for older‐onset patients, there is a significant gap in our understanding of the distinct challenges younger CRC patients may face. This discussion with patient advocates illuminated details of financial and QOL impacts resulting from a CRC diagnosis among early‐onset survivors and raised questions about how these may affect early vs. late onset survivors differently. All the meeting participants commented on the profound financial impacts of a CRC diagnosis. Employment emerged as a dominant theme around which many of their concerns revolved. The ways participants described impacts related to employment can be broken down further into four inter‐related themes: career trajectory, lost earnings, performance and availability of adequate health insurance coverage and benefits. The participants also described how a CRC diagnosis impacted their overall QOL, highlighting both emotional and physical side‐effects of the disease. The emotional side‐effects described were related to overall stress, challenges in their interpersonal relationships and a general lack of information and resources to help them cope with the challenges of disease and survivorship. The physical side‐effects that affected their QOL resulted from both the cancer itself and the subsequent treatment.

Our study is unique in its focus on CRC survivors with early‐onset disease. Most prior studies have reported on financial burden of cancer in general across all age groups.^29^, ^30^ Consistent with our results, prior studies have reported that financial hardship is related to lower QOL.^29^, ^30^ A study of over 2000 cancer survivors (any type of cancer), aged 18‐81, found that financial distress associated with cancer was the strongest predictor of lower QOL. These results may be even more pronounced among younger CRC survivors that experience negative impacts of their career trajectory, earning potential, and health insurance and benefits.^30^ Similarly, Kale, et al linked higher financial burden among cancer survivors with lower physical and mental health scores, and a greater chance of depression and worries about recurrence.^31^

Previous studies also have shown that those diagnosed at younger ages are more susceptible to financial hardships. Zafar et al, found that ‘significant or catastrophic subjective financial burden’ afflicted 42% of cancer patients, with younger patients more likely than older patients to experience financial hardships.^29^ Ramsey, et al, reported that younger cancer patients had higher rates of bankruptcy compared with cancer patients ages 65 or older,^16^ and a survey on financial hardship among 1,202 adult cancer survivors found that financial hardship was more common among those aged less than 65 years compared with those 65 and older.^10^

Research focusing specifically on CRC survivors also finds high risks for financial distress and lower associated QOL, but this work was not focused on early‐onset CRC. According to Regenbogen, et al, CRC patients who experienced post‐surgery complications had significantly higher financial burden than those without, worried more about their finances, were much later to return to work and used more compensation strategies such as taking out loans, spending savings and not making credit card payments.^32^ Others have reported work‐related limitations such as changes in work functioning, attitudes of employers and colleagues, financial pressures and emotional responses.^33^ The advocates in our analysis echoed these findings, emphasizing that their financial hardships were unique to being diagnosed with CRC at a younger age. However, our analysis does not reveal whether the financial impact among these patient advocates is unique to CRC as compared to other cancer types.

Although prior CRC QOL studies have focused on decreased QOL associated with colostomy,^8^ the advocates in this discussion only referred to their ostomy in relation to how it impacted their personal and sexual relationships. The physical side‐effects of colostomy were not discussed in this group, but the emotional impacts were prevalent. Thus, future research should evaluate personal and relationship counselling interventions that help CRC survivors cope with the emotional side‐effects that accompany colostomy, including embarrassment and decreased feelings of intimacy.

### 4.1 Limitations

The limitations of our study should be acknowledged. Participants in this discussion were not representative of all early‐onset CRC survivors. They were members of a CRC‐focused advocacy group, and thus, they are individuals who sought out support and a way to ‘give back.’ They were also all employed and had health insurance at the time of their diagnosis and had the means to incur some travel costs for the advocacy training. It is likely that the financial burdens expressed in this group may be even more pronounced in CRC survivors with less reliable employment, insurance and access to health care. While this group of CRC advocates is not completely representative of all early‐onset CRC patients, this analysis highlights important themes of financial burden and QOL that may be unique to patients diagnosed with CRC under the age of 50 and, thus, warrant further study.

## 5 CONCLUSION

We applied a qualitative analysis to better understand the unique challenges of early‐onset CRC survivors and to identify topics which may warrant further qualitative and quantitative research in larger populations to help health systems provide better cancer care. The advocates exposed important gaps that fall clearly within a traditional scope of health‐care delivery, including a need for more resources to help patients better understand what to expect at different points during the cancer experience. Advocates wanted more information on a wide range of lifestyle topics including how CRC treatment could result in dietary changes, how to cope with changing relationships, how to manage employer expectations and help with managing finances. Some of the emotional and relationship impacts that were mentioned highlight a need for increased social and mental health services to support the patients throughout their survivorship experience. Thus, our results highlight important gaps in the care and support of early‐onset CRC patients. Given the growing incidence of CRC in those under 50,^3^ it is particularly important to address these gaps through additional research and to identify ways to develop care plans that address early‐onset patients' financial, emotional and QOL needs.

## CONFLICT OF INTEREST

The authors declare that they have no conflict of interest.

## Data Availability

The data that support the findings of this study are available upon request from the corresponding author. The data are not publicly available due to ethical or privacy restrictions.
